# Phytochemical Profile and Antioxidant Activity of *Nigella sativa* L Growing in Morocco

**DOI:** 10.1155/2021/6623609

**Published:** 2021-04-20

**Authors:** Salima Tiji, Ouijdane Benayad, Mohamed Berrabah, Ibrahim El Mounsi, Mostafa Mimouni

**Affiliations:** ^1^Electrochemistry Team's Research, Applied Chemistry and Environment Laboratory (LCAE), Faculty of Sciences, University Mohammed First (UMP), Oujda, Morocco; ^2^Solid Mineral Chemistry Team's Research, Applied Chemistry and Environment Laboratory (LCAE), Faculty of Sciences, University Mohammed First (UMP), Oujda, Morocco

## Abstract

**Background:**

*Nigella sativa* L (NS) is a powerful antioxidant and medicinal plant with many therapeutic applications particularly in traditional medicine for respiratory, gastrointestinal, rheumatic, and inflammatory disorders, as well as cancer.

**Objective:**

The aim of this study is to extract the active ingredients from the Moroccan *Nigella sativa* L and determine its antioxidant properties. We hypothesize that the separation of the compounds from *Nigella sativa* L has either a positive or negative effect on antioxidants. To study this, we explored different methods to simultaneously extract and separate compounds from *Nigella sativa* L and performed antioxidant tests (*β*-carotene and DPPH) for all collected fractions.

**Methods:**

*Nigella sativa* L was hot-extracted by Soxhlet and mother extracts and was separated using silica column chromatography with adequate eluents. Qualitative phytochemical tests to determine the chemical families in *Nigella sativa* L seeds were performed on the fractions. They were also identified and characterized by GC-MS and HPLC-DAD. Then, antioxidant activity was examined by *β*-carotene bleaching and DPPH radical scavenger tests. *Results and Conclusion*. The mother extract hexane **FH** generated eight different fractions (**SH1-8**) and the acetone extract **FA** generated 11 fractions (**SA1-11**). The **FH** fractions had a high percentage of fatty acids, and the **FA** fractions had some interesting polyphenols derivative compounds. Phytochemical screening revealed secondary metabolites such as polyphenols flavonoids, alkaloids, steroids, terpenes coumarins, tannins, and saponins. We found that only two solvents (hexane, acetone) of different polarities could easily extract and simultaneously separate the components of *Nigella sativa* L. The antioxidant fractions that we collected had close activity to reference compounds but were more active than the corresponding mother extracts. Moreover, several IC_50_ values of fractions from acetone extract were better than those from hexane. Therefore, the antioxidant activity of *Nigella sativa* L is more attributed to flavonoids and polyphenols than fatty acids. In summary, the separation of hexane extract presents a more pronounced positive effect for antioxidant tests than acetone extract.

## 1. Introduction

Artificial antioxidants such as butylated hydroxytoluene (BHT) and butylated hydroxyanisole (BHA) are commonly reported for their efficiency in delaying cell deterioration but are also suspected to have negative health effects like carcinogenesis and toxicity [1]. Therefore, replacement with natural antioxidants could reduce health risks. *Nigella sativa* (*NS*) is a traditional and natural source of antioxidants [2, 3]. In fact, *NS* is capable of free radical inhibition [4, 5] and can also significantly reduce oxidative stress [6, 7].

*Nigella sativa*, or black caraway, is a medicinal plant with many therapeutic uses in traditional Jordanian folklore. It is used to treat respiratory, gastrointestinal, rheumatic, and inflammatory disorders, as well as cancer [8, 9]. It is also used to treat various respiratory disorders across the globe in Morocco, Pakistan, and Southern Europe [10].

The seeds of *NS* are used as a spice in bread, yogurt, marinades, sauces, and salads. In Islamic culture, *NS* is known as “El Habba Saouda” and is used in traditional medicine with reference to a proverb stating it is “a drug for all diseases except death.” These words remained a mystery, until science was able to determine its therapeutic properties [11].

Considering the richness of its biological heritage, it is possible that the seed extracts could contain one or more active ingredients that specifically target each disease. Indeed, the diversity of secondary metabolites [12–14] can explain this variety of therapeutic uses.

Most previous studies explored the compounds hot- or cold-extracted with polar or nonpolar solvents as well as the essential oil [15, 16]. These extracts contain a mixture of several families of chemical compounds; therefore it is highly unlikely that a single well-defined compound explains all therapeutic uses. In this work, we focus on two essential mother extracts that may contain distinct groups of secondary metabolites.

Hexane is a nonpolar solvent, which can only extract nonpolar compounds such as lipids (fatty acids). On the other hand, acetone is a polar solvent that can extract polar entities such as flavonoids and polyphenols. The use of a solvent of intermediate polarity (chloroform or ethyl acetate) that contains a mixture of the two groups of compounds is likely to inhibit separation. This study aimed to extract and separate these secondary metabolites simultaneously and evaluate their antioxidant activity. To the best of our knowledge, this is the first study to use chromatography to separate secondary metabolites of Moroccan *Nigella sativa*. To do this, we separated the mother extract, with known antioxidant activity, and generated several fractions, which in turn pass the antioxidant tests. Only those fractions exhibiting antioxidant activity moved forward with testing.

Migration of the compounds on TLC plates was used to determine the number of compounds, or groups of compounds, existing in the fraction, and was observed directly on the plates using a UV lamp. The GC-SM and HPLC-DAD analysis was used for compound identification and to explain the antioxidant activity of each fraction.

## 2. Materials and Methods

### 2.1. Chemical Reagents

All solvents and chemical compounds were purchased from Sigma Aldrich. Commercial organic solvents, hexane, chloroform, ethyl acetate, and acetone were of analytical grade (∼99.5%). Chemical compounds (Silica gel, DPPH radical, *β*-carotene) used were of high purity. The phytochemical screenings used were TLC, Liebermann–Burchard, Mayer reagent, Dragendorff, and Folin–Ciocâlteu.

### 2.2. Plant Materials

The *Nigella sativa* seeds L used were harvested in Morocco during the last season and purchased from a local market at Oujda city. They were cleaned and ground in a blender to obtain a fine powder and stored in a dark place for future use.

### 2.3. Extraction

The powder was subjected to extraction using the following solvents: hexane, chloroform, ethyl acetate, and acetone. The extractions took place over 24 hours using a Soxhlet apparatus at 40–50°C. The solvent was then evaporated on a rotavapor (BUCHI Rotavapor R-210) in vacuum rotary at 40°C. The percentage of the extract yield was calculated using the mass of the seeds powder initially placed in the Soxhlet.

### 2.4. Fraction's Separation by Column Chromatography

Only two extracts were chosen for evaluation, those of hexane and acetone origin, due to the time-sensitive nature of separation by column chromatography. These two solvents were chosen because for their significant lipophilia gap; the difference of polarity would allow for simultaneous extraction and separation of compound groups with very varied physicochemical properties. In fact, hexane is a nonpolar solvent which promotes the solubility of hydrophobic compounds, while the polar solvent acetone solubilizes hydrophilic compounds.

Extracts were fractioned by silica gel column chromatography using 20% hexane/80% dichloromethane eluent system for hexane extract and 50% cyclohexane/50% dichloromethane eluent system for acetone extract. The dimensions of column chromatography were 6.5 cm × 47 cm and the porosity of the silica gel was 60A°(63–200 *μ*m).

### 2.5. Identification Analysis

#### 2.5.1. Gas Chromatography Coupled to Mass Spectrometry (GC-MS) Analysis

Fraction investigation was performed on GC-MS using SHIMADZU instrument (GC-MS-QP2010) under computer control at 70 eV. About 1 *μ*l of every fraction was injected into the (GC) column (30 m × 0.25 mm, 0.25 *μ*m) under a flow rate of helium gas equal to 1.4 mL/min. Scanning continued for 28 min and the ionization temperature was maintained at 200°C. The identification and quantification of the compounds were determined by comparing retention indices and spectral mass fragments with computer library NIST147 LIB [17].

#### 2.5.2. High Performance Liquid Chromatography Coupled with Diode Array Detector (HPLC-DAD) Analysis

The identification of phenolic compounds in the acetone extract fractions was carried out on an analytical HPLC using Waters e2695 and diode array detector. Chromatographic separation was performed through a C18 column (5 *μ*m, 250 × 4.6 mm). The solvents composition and the gradient elution process followed Mechraoui et al. [18] with some modifications. The elution was performed by a binary gradient system (A: water/acetic acid (2%v/v) and B: acetonitrile, pH = 2.6). The gradient process was 0–5 min: 95% A and 5% B, then 25–30 min: 65% A and 35% B, 35–40 min: 30% A and 70% B; and 40–45 min: 95% A and 5% B. A flow rate of 0.9 mL/min was used and 30 *μ*L fraction samples were injected after being passed through a 0.45 *μ*m filter. The UV detection was between 280 and 360 nm [18]. Phenolic compounds were identified by comparing retention time and UV-detection relative to the standards.

#### 2.5.3. Phytochemical screening

Phytochemical tests rely on specific revealers for each type of chemical family compound (steroids, alkaloids, and flavonoids). The principle of this method is based on visual observation of color change either by spraying the revealers directly on TLC plates or by adding a few drops of the revealers to the solution. These methods are described in detail by several authors in the literature [19, 20].

Phytochemical screening was performed with thin layer chromatography. TLC plates of silica gel supported by aluminum plates (fluorescent index 254 nm Fluka Analytical, Sigma–Aldrich) and eluents were used to achieve the separation. Different solvent developers were used with the reagents for the chemical screening.

*Steroids/terpenes*. Sterols were identified by Liebermann–Burchard reagent. 50 mL of ethanol was added to 5 mL of acetic anhydride and 5 mL of sulfuric acid on ice. The fraction characterization was performed using TLC plates pulverized at 100°C for 10 min. [19].

*Alkaloids*. Mayer reagent (1 g of plant extract was placed with potassium mercury tetraiodide and water in a test tube) was used for the extraction of alkaloids. Precipitation would occur if the test was positive. Dragendorff reagent: potassium tetraiodobismuthate was pulverized on a TLC plate for fraction separation. Orange spots were detected by UV lamp at 365 nm [21].

*Flavonoids*. A solution of AlCl_3_ and 1% ammonium was pulverized on a TLC plate with the fraction. Yellow spots indicated flavones, blue spots showed phenolic acid, and red spots revealed the existence of Gallic acid [22].

*Saponins*. 2% SbCl_3_ in chloroform solution was pulverized on a TLC plate with the fraction. Red fluorescent spots were detected at 254 nm by a UV lamp [20].

*Polyphenol*s. Polyphenols were identified by Folin–Ciocâlteu reagent. On a TLC plate with the fraction, Folin–Ciocâlteu reagent was pulverized, and the spots were detected at 365 nm by a UV lamp.

*Tannins*. A solution of 2% FeCl_3_ and acetic acid was pulverized on the fractions and placed on a TLC plate. Yellow spots were detected at 366 nm [21].

*Coumarins*. Coumarins were identified by two processes. First, a solution of KOH 2% was pulverized on a TLC plate or by a pulverized solution of 5% (CH_3_CO_2_)Pb on MeOH. The spots were visualized at 365 nm by a UV lamp [21].

### 2.6. Antioxidant Activities

#### 2.6.1. *β*-Carotene Bleaching Test

This method was developed by Sun and Ho [23] and is based on the measure of absorbance at 470 nm due to the decomposition of linoleic acid. An emulsion of *β*−carotene-linoleic acid is prepared with Tween20 and hydrogen peroxide. 2 mg *β*-carotene is dissolved in 10 mL chloroform. 1 mL of this solution is added to 20 *μ*L linoleic acid and 200 mg Tween 40. The chloroform is evaporated at 40°C under vacuum using a rotary evaporator and the residue is taken up to 100 mL by aerated distillated water with vigorous agitation. 4 mL of the emulsion is placed in a series of tubes containing 200 *μ*L samples of the fractions at different concentrations. The tubes are well shaken, and the absorbance is read at once at 470 nm. The tubes are placed at 50°C for 120 minutes with a control, which contained only the emulsion and methanol. Absorbance was recorded and the inhibition percentage was expressed using equation (1). IC_50_ is the 50% inhibition concentration determined graphically on the % I = *f* (*C*) curve by the extrapolation on the *X*-axis for the value of *Y* = 50% inhibition. IC_50_ was determined for the extracts and fractions. For *β*-carotene, the inhibition percentage was calculated according to the following equation:

(1) $$\begin{array}{c} I\%=\frac{AA\left(120\right)\;–\;AC\left(120\right)}{AC\left(0\right)-AC\left(120\right)}\times 100, \end{array}$$

where **AA** (120) is the absorbance at 120 min; **AC** (120) is the control absorbance at 120 min; and **AC** (0) is the control absorbance at 0 min.

#### 2.6.2. DPPH Radical Scavenging Activity

DPPH radical activity was carried out by the Gramza-Michalowska method [24]. DPPH is a stable radical, which has a free electron on the nitrogen atom. In the presence of antioxidant DPPH, the color changes from violet to yellow [25]. In this test, various concentrations of fractions were added to the methanol solution of DPPH radicals (0.004% m/v). The samples were incubated for 30 minutes at room temperature and the absorbance was recorded at 517 nm against a blank solution. The percent inhibition was calculated using equation (2). The inhibitory concentration (IC_50_) is determined as the concentration that inhibit 50% of DPPH (I% = 50). The inhibition percentage is calculated using the formula below:

(2) $$\begin{array}{c} I\%=\frac{\left(Ac\;–\;Asample\right)}{Ac}\times 100, \end{array}$$

where **Ac** is the absorbance of DPPH without antioxidant (negative control) and **Asample** is the absorbance of DPPH in the presence of an antioxidant.

## 3. Results and Discussion

### 3.1. Extraction's Yield

In the successive extraction of the *Nigella sativa* L seeds (*NS*), the yields of mother extracts (FH and FA) were calculated relative to the mass of the starting powdered seeds placed in the Soxhlet apparatus. Hot extraction is more beneficial than cold extraction because the maceration and provides significantly higher yields [26]. In general, the color of the mother extracts obtained is more or less deep brown. The results show that the mother hexane extract, **FH**, is the most abundant (34.2%), while the acetone mother extract, **FA**, only represented 2%. It can be underlined that *NS* contains essential fatty acid compounds (about 30%). The final residue devoid of its chemical composition and probably consisting of fibers exhibits a 45.6% yield. This yield is similar to those of Khoddami et al. [27] (37.33%) and Matthaus [28] (36%.) However, Antuono et al. [28] obtained only 26% of *NS* in Moroccan seeds. Similarly, Sun and Ho [23] obtained comparative acetone extract yields at 2.5%.

### 3.2. Hexane and Acetone Extracts Separation

Only two promising mother extracts from hexane **FH** and acetone **FA** were separated by silica gel chromatography. Separation was slow and fractions were collected every five min and systematically assessed on TLC plates.

Table 1 shows the yield of assembled fractions in relation to their respective mother extract. The yields were calculated relative to the mass of the mother extracts **FH** and **FA** used at the beginning. Following this procedure, the hexane mother extract generated eight fractions, **SH1-8**, and that for acetone fractions there were 11 fractions, **SA1-11**.

The eight fractions generated by hexane (Table 1) show varying yields with the highest being **SH1** with 72.35% followed by **SH7** (9.11%). The others give yields less than 5.12%. The yields of fractions generated by acetone extract were calculated relative to the mass of the mother extract of acetone (**FA**) introduced at the beginning. **SA1** represents the highest yield at 62.88% followed by **SA11** at 15.82%. The other fractions were all less than 10%.

### 3.3. GC-MS Analysis

#### 3.3.1. Hexane Extract

**Figure 1:**
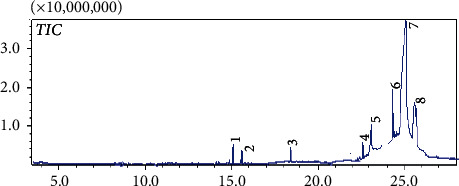
Gas chromatogram of the mother hexane extract **FH**.

The results of **FH** characterization by GC-MS are presented in Figure 1 and Table 2. The recorded chromatogram after the extraction (Figure 1) shows the eminent presence of eight compounds. In Table 2, the chemical composition of 85.9% is identified as linoleic, palmitic, Hexadecanoic, and oleic acids.

We noted that, according to Table 2, the seeds from eastern Morocco mainly contain linoleic acid (80.26%) and much less palmitic and oleic acid. This is in line with the work of several other authors [10, 27, 29–31].

The presence of other compounds was less than 6.24%, but it should be noted that the majority of the compounds extracted had similar structures. It was also observed that the rest of the compounds extracted differed in the literature. This can be explained by spontaneous chemical reactions occurring as a result of physical conditions (i.e., light and temperature) without any experimental intervention.

In Morocco, Gharby et al. [29] reported 58.5% of oleic acid after extraction of esterified hexane extract, while Khodami et al. [27] showed that hexane extract of *NS* was mainly unsaturated fatty acids such as palmitic, linoleic, and oleic acids. Piras et al. [30] reported that Turkish *NS* contained 55% unsaturated fatty acids. Unidentified components in the esterified hexane extract represented only 13%. Those might be dependent on the esterification/methylation process. Ekowati et al. [31] and De Oliveira and Khater [10] reported many other compounds not found in our work.

#### 3.3.2. Fractions of Hexane Extract

The identification results of fractions **(SH1-8)** from **FH** by GC-MS analysis are presented in Table 3. The results indicate the presence of a considerable variety of compounds. We observed that the majority were fatty acids.

Several constituents were present in different fractions (e.g., palmitic acid identified in fractions **SH1**, **SH2**, **SH3**, **SH5**, **SH6**, **SH7**, and **SH8;** linoleic acid in **SH1**, **SH2**, **SH5**, and **SH7** and oleic acid found in fractions **SH3**, **SH6**, and **SH8**. The appearance of fatty acids in many fractions could be explained by the disturbance of compounds migrating in the column because of the abundance of fatty acids. In fact, a high quantity of fatty acids and their derivatives migrated poorly in the column chromatography. To overcome this problem, we proceeded to centrifugation and then cooled the extract to −4°C to separate the solid fat. Only 1.5% of the fatty acids were eliminated using this method.

In general, naturally extracted plant products are continuously transformed by oxidation or other chemicals reactions. Consequently, their percentage varies indefinitely despite good conservation. For example, olive oil retains its physicochemical characteristics for a whole year, but loses taste quality over time [32]. The variation of the quantity and quality of the hexane extract and fractions during migration in the chromatography column is justified. In addition, the chromatography column is slightly acidic, and the eluting solvents contain traces of transition metals, which can cause additional chemical reactions and transformations.

### 3.4. HPLC-DAD Analysis

**Figure 2:**
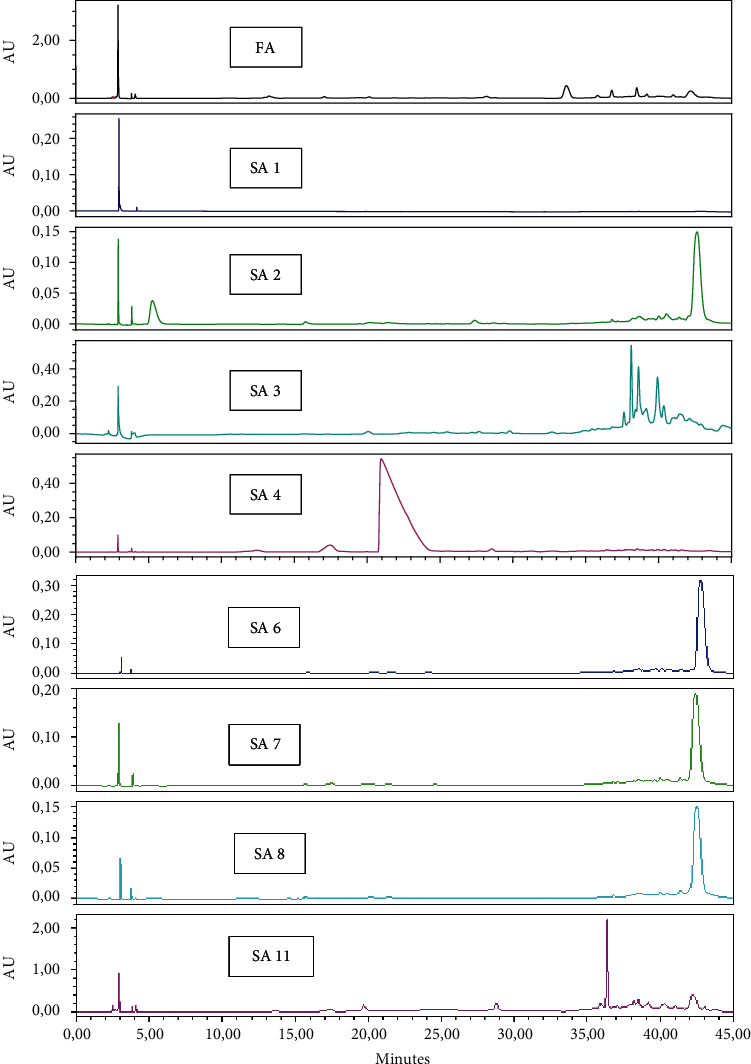
HPLC chromatograms fractions generated from the acetone mother extract **FA**.

The results of the HPLC analysis are presented in chromatograms (Figure 2) that show the constituents of the acetone extract and its fractions, while Table 4 shows the identified constituents. On the *Y*-axis, the values of the peak's intensities are arbitrary. We can compare the intensities of the peaks on the same scheme, but we cannot compare them with the others because the collected fractions were concentrated each time by evaporation in a rotavapor. Therefore, we can only compare the position of the peaks in the chromatogram on the *X*-axis.

The acetone extract fractions gave different chromatograms compared to the hexane fractions. The peaks showed varying intensity depending on their abundance (Figure 2).

The **FA** contained a mixture of several compounds. Gallic acid is the major compound in Figure 2. It is also clearly visible in the **SA1**, **SA2**, and **SA3** fractions. Our results are in agreement with those reported by Mechraoui et al. [18]. Except the kaempferol, all compounds found in the mother extract disappeared in the separated fractions. It should be noted that the separation was successful since, at the end, each fraction contained only one (**SA1**, **SA4**), two (**SA3**, **SA7**), or three (**SA2**, **SA11**) compounds. Due to technical problems, some fractions (**SA5**, **SA6**, and **SA8**) could not be analyzed and identified.

### 3.5. Phytochemical screening

Tables 5 and 6 summarize the results of the phytochemical screening for all hexane and acetone extract and fractions. The presence of phytochemical components of steroids, terpenes, coumarins, alkaloids, polyphenols, flavonoids, and saponins was determined.

The hexane mother extract had steroids, terpenes, tannins, saponins, and alkaloids (Table 5). However, coumarins, polyphenols, and flavonoids were absent. The acetone mother extract only contained polyphenols, steroids, tannins, flavonoids, and alkaloids (Table 6), while coumarins and saponins are not present.

Most fractions had alkaloids and saponins as well as several secondary metabolites like steroids and terpenes. Coumarins, polyphenols, and flavonoids appeared in some fractions despite their absence in the original extract (Table 5). This can be explained either by the phenomenon of opacity, where certain colors can be concealed by others, or by disparity of the color's wavelengths during measurements.

The phytochemical results of the *NS* hexane extract are similar to those reported by Javed [33], except in the absence of sterols in their hexane extract. Other phytochemical investigation [34] described close phytochemical screening to our results. Nevertheless, coumarins are reported and saponins were absent in our results.

Contrary to the hexane mother extract, the compound families in the acetone mother fraction were the same as those in the separation fractions (Table 6). The presence of polyphenols and flavonoids is marked in several fractions, and less for other chemical families. In contrast, some authors report that acetone extract has a high level of steroids and are low on coumarins [34].

### 3.6. *β*-Carotene Bleaching Test

Table 7 presents the values of the inhibitory concentrations necessary to reduce 50% oxidation (IC_50_) by the antioxidant *β*-carotene. The values are given for both hexane and acetone mother extracts, as well as their corresponding fractions, which are compared with the reference value of butylhydroxyanisole (**BHA**). At the beginning, the quantity collected from certain fractions was extremely low. Hence, we could not perform totality tests of all fractions without exhausting our stock. All hexane fractions were more active than their mother extract **FH**; however the acetone mother extract **FA** was more active than three of its fractions.

The activity of fractions from the hexane extract was dispersed as follows: **SH4** > **SH1** > **SH7** > **FH**. **SH4** had the highest activity and resemblance to the reference **BHA** (Table 7), likely due to the presence of steroids and terpenes. Although **SH1** and **SH7** also contained steroids and flavonoids, they had a weak activity compared to **SH4**. This can be attributed to the presence of other families of chemical compounds, which inhibit the apparent activity.

The characterization by GC-MS showed the existence of many antioxidant components [35], which have similar structure to **BHA**. In addition, derived terpenes were identified, such as 2.4-decadienal and (Z) 9-Tricosene. Moreover, fatty acid analogues, like linoleic acid, 17 pentatriacontene, or 2.4-decadienal, were also found. To support this claim, **SH4**, **SH1**, and **SH7** were compared to vegetal species with high antioxidant activity like sage, thyme, and curcuma [35, 36]. In the extract's lipids, the present antioxidant and anticancer phytosterols were also detected [37, 38].

In acetone extract and fractions, we observed that the inhibition activity is dispersed as follows: **SA5** > **SA10** > **FA** > **SA2** > **SA1** > **SA4**. The first group of fractions composed of **SA5** and **SA10** had a much higher activity than **FA** with IC_50_ remarkably close to **BHA** (Table 7). The second group composed of **SA2**, **SA1**, and **SA4** exhibited weaker antioxidant activities than **FA**.

Singh et al. [39] reported that **FA** was the most interesting part of *NS* because it has the highest antioxidant activity. Mechraoui et al. [18] found that, in *β*−carotene tests, the IC_50_ from the acetone extract was 0.2444 mg/mL. The IC_50_ value of pure gallic acid found by Lu and Khoo [40] (10.50 *µ*g/mL) was different from that which we found in **SA1** containing exclusively gallic acid. The IC_50_ for rutin is estimated to be 26.2 mg/mL by Yang et al. [41]. The only pure flavonoids like apigenin, quercetin, and naringenin gave interesting antioxidant activity [42, 43]. This result can be extrapolated to the results found for fractions **SA4**, **SA5**, and **SA10** since they only contain these flavonoids.

### 3.7. DPPH Radical Scavenging Activity

The values of (IC_50_) determined by the DPPH antioxidant tests are summarized in Table 8. They were carried out for the hexane and acetone mother extracts, as well as their corresponding fractions, which were all compared with the reference value of ascorbic acid. IC_50_ of the hexane extract was the highest and the acetone mother extract was significantly lower than four of its fractions.

In the case of hexane, the **SH7** and **SH8** were more active than **FH**, particularly **SH8** whose IC_50_ was three times less. The antioxidant activity increases as follows: **SH8** > **SH7** > **FH**.

In the case of acetone, the activity value of the mother extract was intermediate between all fractions; the antioxidant activity increases in the direction **SA11** > **SA5** > **SA10** > **FA** > **SA2** > **SA4** > **SA1** > **SA3**. The three most active fractions, **SA11**, **SA5**, and **SA10**, all had an IC_50_ lower than **FA** but not comparable to the reference ascorbic acid.

Considering the two antioxidant tests, *β*-carotene, and DPPH^●^, the fractions from the acetone extract were more active than those from the hexane extract. For *β*-carotene, the best value was found in the acetone fraction **SA5** with IC_50_ = 0.064 mg/mL, which is remarkably close to the BHA reference, which has a value of IC_50_ = 0.053 mg/mL. In the case of DPPH, all the acetone fractions showed acceptable IC_50_ values, particularly fractions **SA5**, **SA10**, and **SA11** which had mean IC_50_ values around 0.25 mg/mL. However, they are extremely far from the reference ascorbic acid (0.018 mg/mL).

Haroun [15] reported that *NS* hexane extract from two different places in the same country (Malaysia) gave IC_50_ = 8.17 mg/mL for *NS* harvested in Shah Alam and IC_50_ = 4.48 mg/mL for *NS* harvested in Kentalan. In Yemen, DPPH test showed an IC_50_ = 12.79 mg/mL, in Sudan it was equal to 4.48 mg/mL [15], and in Ethiopia IC_50_ was 8.52 mg/mL. Soulaimanifar et al. [15] reported a very high IC_50_ value, 104.76 mg/mL, in Iran. In Tunisia, Ksouda et al. [44] reported that *NS* hexane extract had IC_50_ = 31 mg/mL, a very close value to our 23.25 mg/mL. The origin of the seeds and the extraction method are key factors for the quality and quantity of secondary metabolites. In Malaysia, the DPPH test on hexane extract by supercritical fluid extraction gave an IC_50_ = 1.58 mg/mL [45].

**SH7** antioxidant activity could be explained by its phytochemical analysis due to the presence of terpenes, steroids, polyphenols, and flavonoids in the fraction. Furthermore, GC-MS analysis gave diene functional groups and unsaturated fatty acids, which are known by their antioxidant activity. Palmitic acid is reported to have an antioxidant effect [46] on *β*−carotene test [47, 48]; polyphenols derivatives like benzoic acid can also inhibit oxidation [47, 49]. **SH7** and **SH8** showed similar chemical compositions with fatty acids, and this may explain a part of their antioxidant activities.

The antioxidant activity of **FA** could be attributed to its flavonoids. HPLC-DAD identified gallic acid, hydroquinone, apigenin, naringenin, ascorbic acid, cysteine, rutin, quercetin, and kaempferol. Mechraoui et al. [18] reported an IC_50_ = 0.1602 mg/mL from Tunisian *NS*. IC_50_ was 2.69 mg/mL from a Pakistani *NS*. These results are different from ours with an IC_50_ equal to 0.79 mg/mL.

The chemical structure of the fractions was identified by HPLC-DAD and the fraction's antioxidant activity was compared to commercial pure compounds. Pure commercial gallic acid IC_50_ was reported as 1.50 *µ*g/mL [40], apigenin IC_50_ was 30.3 *µ*g/mL, rutin had an IC_50_ = 23.7–50 *µ*g/mL [41, 50], and quercetin presented with an IC_50_ = 1 *µ*g/mL [40]. These products are the essential compounds in fractions **SA1**, **SA2**, and **SA11**. It is clear that fractions of a single compound and fractions with multiple compounds do not have the same antioxidant activity level. There can be a positive synergistic effect on the activity or a negative one (inhibitor effect). If we compare the activity of **SA1** and **SA3**, we can see that catechol in the presence of gallic acid has a negative effect on the antioxidant activity, whereas, in the case of **SA2**, hydroquinone and apigenin had a positive effect on activity. Therefore, testing is required to be able to predict the synergistic effects.

## 4. Conclusion

This study describes the simultaneous process of extraction-separation of chemical components of *Nigella sativa* L seeds based on the polarity of two well-chosen solvents. We have succeeded in extracting and separating two families of compounds with different physicochemical and phytochemical properties. Analysis and separation methods like GC-MS and HPLC-DAD allowed us to characterize and identify the majority of the mother extracts and separated fraction constituents. It should be noted that the Moroccan *Nigella sativa* L is rich in linoleic acid but low on oleic or palmitic acid.

Antioxidant activity was evaluated by *β*-carotene bleaching and DPPH scavenger. Antioxidant activity was attributed to each fraction and explained by its constituents. *Nigella sativa* L contains important secondary metabolites and is as a reliable source of antioxidant compounds. Moroccan *NS* gives a competitive IC_50_ compared to several countries around the world.

We show that secondary metabolite separation is beneficial for antioxidant activity. In fact, we repeatedly found that the fractions were more active than the mother extracts. In addition, some of the fractions had an underlined activity compared to the references.

We have succeeded to identify some of the interesting fractions generated by the mother extract and specified the compounds responsible for antioxidant activities. In fact, it can be said that, for *β*-carotene test, antioxidants are concentrated in hexane fractions **SH4**, **SA5**, and **SA10**. For DPPH test, fractions **SH8**, **SA5**, **SA10**, and **SA11** include the active entities of *Nigella sativa* L. Future research should explore the active fractions *in vitro* and compare their antioxidant activity in animal models before and after induction of diseases caused by oxidative stress.

## Figures and Tables

**Table 1:** Yields of fractions derived from the mother extracts of hexane **FH** and acetone **FA**.

Hexane		Acetone	
Fraction	Yield/FH (%)	Fraction	Yield/FA (%)
**FH**	100	**FA**	100
**SH1**	72.35	**SA1**	62.88
**SH2**	3.45	**SA2**	9.92
**SH3**	0.40	**SA3**	3.30
**SH4**	0.67	**SA4**	3.55
**SH5**	5.12	**SA5**	0.15
**SH6**	1.34	**SA6**	2.31
**SH7**	9.11	**SA7**	1.16
**SH8**	4.3	**SA8**	0.42
		**SA9**	0.44
		**SA10**	0.05
		**SA11**	15.82

**Table 2:** Characterization by GC-MS of the chemical composition of the mother hexane extract of NS. TR: retention time; %Air: percentage of compounds present.

N°	Names	This study		Composition of references				
		TR	% Air	[29]	[30]	[27]	[31]	[10]
1	2.4-Decadienal	15.10	1.79				1.2	
2	2-oxo-méthyl ester Hexadecanoic acid	15.59	1.06					
3	Phenol. 4-methoxy-2.3.6-trimethyl-	18.41	1.56					
4	Palmitic acid-methyl ester	22.60	1.32	13.1	12–13%	14.11	1.9	
5	Ascorbic acid 2.6-dihexadecanoate	23.11	4.39					
6	Oleic acid methyl ester	24.36	2.96	23.8	22%	21.25		**+**
7	Linoleic acid	25.12	80.65	58.5	54–55%	56.71	67.59	**+**
8	E.Z-1.3.12-Nonadecatriene	25.61	6.24					
	Stearic			2.3	2–3%			
	9.12-Octadecadienoic acid (Z.Z)/cis linoleic acid						10.18	
	(9E.12E)-9.12-Octadecadienoic Acid						4.2	
	9.12-heptadecadienoate						4.56	
	(9Z)-9.17-Octadecadienal						2.3	

+Presence.

**Table 3:** Fraction chemical composition generated from the hexane mother extract and identified by GC-MS.

Fraction	Pic number	Names	TR	%Air
SH1	1	2.4-Decadienal	15.11	4.95
	2	lauric acid	18.68	1.87
	3	Palmitic acid	22.61	8.64
	4	17-bromopropanoic acid	23.26	9.3
	5	1-Octadecanol	23.58	17.79
	6	Linoleic acid	24.36	18.29
	7	Heptadecanoic acid	24.64	11.02
	8	9-Octadecanoic acid (Z). 2-butoxyethyl ester	25.79	28.12

SH2	1	Octanoic acid. 8-hydroxy-	16.23	2.61
	2	Methyl azelaaldehydate	16.77	2.60
	3	Azelaic acid	18.26	9.44
	4	Laural dimethyl acetal	18.89	12.14
	5	Palmitic acid	22.62	40.98
	6	Linoleic acid	24.37	17.10
	7	8-Octadecenoic acid	24.43	1.25
	8	Heptadecanoic acid	24.62	16.35

SH3	1	2.4-Decadienal	15.24	1.70
	2	Palmitic acid. Methyl ester	22.60	5.65
	3	1-Octadecanol	23.84	87.32
	4	Oleic acid. Methyl ester	24.34	5.31

SH4	1	Hexadecane. 7.9-dimethyl-	15.17	9.97
	2	Hexadecane	18.13	10.84
	3	Tricosanoic acid10.14.18.22-tetramethyl	22.58	11.17
	4	17 pentatriacontene	24.59	60.37
	5	Lignocerol	24.79	7.62

SH5	1	Hexadecane. 2.6.11.15-tetramethyl-	17.55	0.29
	2	9-Eicosene. €-	18.78	1.79
	3	Palmitic acid. Methyl ester	22.60	2.65
	4	9-Tricosene. (Z)-	23.28	2.70
	5	linoleic acid	24.05	92.54

SH6	1	Eicosane	18.13	2.19
	2	Dichloroacetic acid.heptadecyl ester	18.77	4.08
	3	9-Dicosene	21.14	5.12
	4	Palmitic acid. Methyl ester	22.60	4.09
	5	l-(+)-Ascorbic acid 2.6-dihexadecanoate	23.03	14.37
	6	9-Tricosene	23.28	6.60
	7	1-Octadecanol	23.91	47.22
	8	Oleic Acid	24.81	12.34
	9	9-Hexacosene	25.24	3.95

SH7	1	Hexadecane. 7.9-dimethyl-	15.18	6.11
	2	Heptadecane. 8-methyl-	15.18	3.71
	3	Hexadecane. 2.6.11.15-tetramethyl-	17.55	8.44
	4	Pentanoic acid. 5-hydroxy-. 2.4-di-t-butylphenyl esters	17.83	3.87
	5	Heptadecane. 2-methyl-	18.13	11.68
	6	9-Eicosene. €-	18.78	16.82
	7	Dichloroacetic acid.heptadecyl ester	21.15	6.42
	8	Palmitic acid. Methyl ester	22.60	18.89
	9	9-Tricosene. (Z)-	23.28	9.74
	10	linoleic acid	24.35	14.08

SH8	1	2.4-Decadienal	15.16	3.82
	2	9-Eicosene. €-	18.77	16.53
	3	Dichloroacetic acid.heptadecyl ester	21.14	16.84
	4	Palmitic acid. Methyl ester	22.60	24.91
	5	1-Octadecanol	23.58	13.23
	6	Oleic acid. Methyl ester	24.39	5.57
	7	Stearic acid. Methyl ester	24.62	10.49
	8	9-Hexacosene	25.23	8.59

TR: retention time; %Air: percentage of compounds present.

**Table 4:** Characterization by HPLC-DAD of fractions chemical compounds generated from the acetone mother extract **FA**.

	Sample	Compounds
1	FA	Gallic acid
		Hydroquinone
		Apigenin
		Naringenin
		Ascorbic acid
		Cysteine
		Rutin.
		Quercetin
		Kaempferol

2	SA1	Gallic acid

3	SA2	Gallic acid
		Hydroquinone
		Apigenin

4	SA3	Gallic acid
		Catechol

5	SA4	Naringenin
6	SA5	ND

7	SA6	ND

8	SA7	Ascorbic acid
		Cysteine

9	SA8	ND

10	SA11	Rutin
		Quercetin
		L-Histidine

ND: not done.

**Table 5:** Phytochemical screening of the hexane mother extract **FH** and its fractions.

Fractions	Steroids& terpenes	Coumarins	Alkaloids	Tannins	Polyphenols	Flavonoids	Saponins
FH	+++	−	+	++	−	−	++
SH1	+	++	+++	−	+++	+	−
SH2	−	+	+	−	++	−	−
SH3	+	−	−	−	−	−	++
SH4	+	−	−	−	−	+	−
SH5	−	−	−	−	−	+	++
SH6	+	−	−	++	−	−	−
SH7	+	+++	−	++	++	++	−
SH8	−	−	−	−	−	+	++

(−) absence; (+) low presence; (++) moderated presence; (+++) high presence.

**Table 6:** Phytochemical screening of the acetone mother extract **FA** and its fractions.

Fractions	Steroids& terpenes	Coumarins	Alkaloids	Tannins	Polyphenols	Flavonoids	Saponins
FA	++	−	+	++	+++	+	−
SA1	−	−	−	−	+	+	−
SA2	−	−	−	−	++	+	−
SA3	−	−	−	−	+++	++	−
SA4	−	−	−	−	++	+	−
SA5	−	−	−	−	+	++	−
SA6	−	−	+	+	−	−	−
SA7	+	−	−	−	−	−	−
SA8)	+	−	−	+	−	−	−
SA9	+	−	−	−	−	+	−
SA10		−	−		+	+	−
SA11	++	−	++	+	+	+	−

(−) absence; (+) low presence; (++) moderated presence; (+++) high presence.

**Table 7:** Antioxidant activity, *β*-carotene bleaching test of mother extracts of hexane and acetone, and their fractions.

Reference	Solvent	*β*-Carotene bleaching test							
**BHA**	**Hexane**	**FH**	**SH1**	**SH4**	**SH7**	**SH8**	-	-	-
**IC**_**50**_ mg/mL	**IC**_**50**_ mg/mL	56	13.2	1.02	14.1	--	-	-	-
**0.053**	**Acetone**	**FA**	**SA1**	**SA2**	**SA3**	**SA4**	**SA5**	**SA10**	**SA11**
	**IC**_**50**_ mg/mL	3.72	7.36	4.46	--	10.04	0.064	0.216	--

**Table 8:** Antioxidant activity of DPPH for hexane and acetone mother extracts and their fractions.

Reference	Solvent	DPPH^●^ test							
Ascorbic acid	**Hexane**	**FH**	**SH7**	**SH8**			-	-	-
**IC**_**50**_ mg/mL	**IC**_**50**_ mg/mL	23.25	16.01	8.7			-	-	-
**0.018**	**Acetone**	**FA**	**SA1**	**SA2**	**SA3**	**SA4**	**SA5**	**SA10**	**SA11**
	**IC**_**50**_ mg/mL	0.79	3.84	1.12	16.8	1.91	0.28	0.31	0.23

## Data Availability

The data used to support the study are included within the article and are also available from the corresponding author.

## Abbreviations

NS: *Nigella sativa*
**FH**: Hexane extract
**FA**: Acetone extract
**SH1-8**: Fractions separated from hexane extract
**SA1-11**: Fractions separated from acetone extract
GC-MS: Gas chromatography coupled to mass spectroscopy
HPLC-DAD: High performance liquid chromatography coupled to diode array detector.
